# Model Independent MRE Data Analysis

**DOI:** 10.1155/2013/912920

**Published:** 2013-04-04

**Authors:** Kogo Yoshikawa, Gen Nakamura

**Affiliations:** ^1^Hokkaido University, Sapporo 060-0810, Japan; ^2^Inha University, Incheon 402-751, Republic of Korea

## Abstract

For the diagnosing modality called MRE (magnetic resonance elastography), the displacement vector of a wave propagating in a human tissue can be measured. The average of the local wavelength from this measured data could be an index for the diagnosing, because the local wave length becomes larger when the tissue is stiffer. By assuming that the local form of the wave is given approximately as multiple complex plane waves, we identify the real part of the complex linear phase of the strongest plane wave of this multiple complex plane waves, by first applying the FBI transform (Fourier-Bros-Iagolnitzer transform) with an appropriate size of Gaussian window and then taking the maximum of the modulus of the transform with respect to the Fourier variable. The real part of the linear phase is nothing but the real inner product of the wave vector and the position vector. Similarly the imaginary part of the linear phase describes the attenuation of the wave and it is given as a real inner product of a real vector and the position vector. This vector can also be recovered by our method. We also apply these methods to design some denoising and filtering for noisy MRE data.

## 1. Introduction

A new measurement modality called MRE (magnetic resonance elastography) consists of an MRI (magnetic resonance imaging), mechanical vibration system, and an additional MRI pulse sequence called MSG (motion sensitizing gradient) synchronized with the time harmonic vibration generated by the vibration system. Given a time harmonic external vibration generated by the vibration system to a human body which yields a wave in the human body, MRE gives a snapshot of the displacement vectors of the wave over each slice of the human body. We call this snapshot MRE data. The slice can be the cross section of the body by any one of the *x*_1_-*x*_2_ plane, *x*_2_-*x*_3_ plane, and *x*_3_-*x*_1_ plane, where (*x*_1_, *x*_2_, *x*_3_) is the Euclidean coordinates. If we can recover the stiffness of the tissue in a human body from the MRE data, MRE can provide a realization of doctors' palpation inside human bodies which has been dreamed about by all the doctors for many years (cf. [1, 2]). We call any procedure to recover the stiffness or extract any information about the stiffness MRE data analysis.

There are two kinds of MRE data analysis. The one is the model-independent MRE data analysis which only assumes that any local wave forms of the wave are given approximately as multiple complex plane waves and recover the real part of the complex linear phase of the strongest wave in this multiple complex plane waves which can be represented by the so-called wave vector. We call this wave vector divided by the angular frequency of vibration the *local wave vector* of the multiple complex plane waves. The other is the model-dependent MRE data analysis which considers some partial differential equation to describe the wave and stiffness as its solutions and coefficient, respectively, and recover the coefficient from the MRE data via this equation. We will call such a partial differential equation the PDE model. In this paper we will give a model-independent MRE data analysis based on the FBI transformation (Fourier-Bros-Iagolnitzer transform). For the model-dependent MRE data analysis see, for instance, [2–6] and the references therein.

It is well known that the wave length becomes larger if the tissue becomes stiffer. In terms of the wave vector this means that the wave vector becomes shorter if the tissue become stiffer. Hence, by looking at the wave vectors in the tissues, we can qualitatively know a change of their stiffness. Since the modeling error is always a big problem in the MRE data analysis, the model-independent analysis has some advantage if it is not so important to recover the stiffness quantitatively but qualitatively.

In the rest of this section we will explain more precisely about our model-independent MRE data analysis. Since the wave length of the longitudinal wave in human tissue is too long to be observed, we can only observe shear waves when the tissues is isotropic tissues and quasi-shear waves if the tissues is anisotropic. Suppose that a shear wave or quasi-shear wave is mainly propagating toward the *x*_2_ direction and we are looking this wave over a slice parallel to the *x*_1_-*x*_2_ plane which is the cross section of a human body. Then, let *φ* = *φ*(*x*_1_, *x*_2_) be the one of the component of displacement vector of this wave perpendicular to *x*_2_ direction, say *x*_3_ component. We also take such a wave whose phase of the vibration is 90 degrees advanced and denotes its component similar as before by *ψ* = *ψ*(*x*_1_, *x*_2_). For our data analysis, it is more convenient to consider

(1) $$\begin{array}{c} u=u\left(x\right)=\varphi \left(x\right)-i\psi \left(x\right) \end{array}$$

than considering *φ* and *ψ* separately.

A naive way of looking at *u* near a point *p* in the cross section is that it is locally given by a finite linear combination of the complex plane wave *ae* ^*ω*(*α*+*iβ*)·(*x*−*p*)^ with an amplitude *a* ∈ *ℂ*, vectors *α*, *β* ∈ ℝ^2^ which do not depend on *x* = (*x*_1_, *x*_2_), and the angular frequency *ω*/(2*π*) of the vibration system. Note that *α* and *β* describe the attenuation and propagation direction of the wave *u*, respectively. We call this form of *u* the *local single-wave form* if the linear combination consists of just one term and *local multiple-wave form* if otherwise.

Let *u* be described approximately as the local multiple-wave form near a point *p* in a region of interest (ROI) of a human tissue. Then, by our method called LWV method (local wave vector method) and LAV method (local attenuation vector method) which are based on the FBI transformation, we can recover *β* and *α* in the strongest local single-wave form of the local multiple-wave form. We will call these *β* and *α* in this strongest local single-wave form the *local wave vector* and *local attenuation vector*, respectively. Here the FBI transformation is a weighted Fourier transformation with the Gaussian window centered around *p*. Once we have recovered *β* at several points in the ROI, we can filter the wave fields with many waves interfering with each other in the ROI to a single major wave. If the ROI is located near the boundary of tissue, for instance the boundary between a tissue and organ, there is an interference of incoming waves and reflected waves from the boundary. In such a place of ROI the wave length and amplitude of wave could become smaller than the other parts of the ROI and hence the profiles of the distribution of the local wave vectors there will become quite complicated. But by our filtering method based on the LWV method, we can extrapolate the major wave up to the boundary in this ROI. As a consequence, we can get very clear filtered wave image having just a major wave in this ROI. We call this denoising method the LWV* denoising* of wave.

To transform the recovered local wave vector *β* and local attenuation vector *α* (Figure 16) to the stiffness of tissue, we need to have a PDE model. Suppose that our tissue can be considered as nearly incompressible isotropic viscoelastic medium, then the above *u* can be considered approximately as the *x*_3_ component of *rot*⁡*v*, where *v* denotes the displacement vector of the wave and *rot*⁡*v* denotes the rotation of *v*. Then, each local single-wave form *u*′ of the local multiple-wave form *u* should satisfy

(2) $$\begin{array}{c} \left(\rho \omega^{2}+\left(G^{\prime }+iG^{\prime \prime }\right)\Delta \right)u^{\prime }\left(x\right)=0 \end{array}$$

approximately in a small neighborhood of *p* with the density *ρ* ≈ 10^3^ kg/m^3^, the storage modulus *G*′, and loss modulus *G*′′. We remark here that *G*′, *G*′′ can change from one region to another region where the local multiple-wave form of *u* changes. Further, we remark that *u*′ always satisfies (2) approximately, if the tissue is modeled as whichever type of nearly incompressible isotropic viscoelastic media [3, 7]. Suppose that we have identified *β* and *α* in the strongest local single-wave form *u*′ of *u*. Then, by substituting this local single-wave form into (2), we have

(3) $$\begin{array}{c} \rho +\left(G^{\prime }+iG^{\prime \prime }\right)\left(\alpha +i\beta \right)\cdot \left(\alpha +i\beta \right)=0 \end{array}$$

which immediately implies that *G*′, *G*′′ are given by

(4) $$\begin{array}{c} \left(\begin{array}{c} G^{\prime }\\ G^{\prime \prime } \end{array}\right)=\frac{\rho }{{\left({\left|\alpha \right|}^{2}-{\left|\beta \right|}^{2}\right)}^{2}+4{\left(\alpha \cdot \beta \right)}^{2}}\left(\begin{array}{c} {\left|\beta \right|}^{2}-{\left|\alpha \right|}^{2}\\ 2\alpha \cdot \beta \end{array}\right). \end{array}$$

Hence (4) gives the link between *α*, *β* and *G*′, *G*′′.

The rest of the paper is organized as follows. In Sections 2 and 3 we give the theories of the LWV method and LAV method, respectively. Then, in the succeeding section we will provide some numerical results for these two methods. Especially, in order to see the effectiveness of these method, we tested our methods by recovering *G*′, *G*′′ of a phantom made of PAAm gel by the MRE group in our university (Professor J. Gong, Laboratory of Soft and Wet Matter, Hokkaido University) and for a phantom made of agarose gel by Mayo Clinic so that we can compare our results with the other results obtained by different MRE data analysis. In the final section, we will apply our methods to the denoising and sharpening of the MRE data. Before closing this introduction, we would like to acknowledge Mayo Clinic providing us the data and emphasize that Mayo Clinic is the front runner of the MRE study.

## 2. LWV Method

In this section we will give the details of the LWV method mentioned in the introduction. Let *W*(*u*; *p*, *σ*)(*ξ*) be the two dimensional FBI transform (cf. [8]) of a locally integrable function *u* in ℝ^2^ with the Gaussian window of size *σ* localized around *p* ∈ ℝ^2^ as follows:

(5) $$\begin{array}{c} W\left(u;p,\sigma \right)\left(\xi \right)=\int_{{\mathbb{R}}^{2}}e^{-ix\cdot \xi }u\left(x\right)e^{-{\left|x-p\right|}^{2}/2\sigma^{2}}dx\;\left(\xi \in {\mathbb{R}}^{2}\right) \end{array}$$

provided that this integral converges which is the case for the local multiple-wave form *u*. This transformation is also called the two dimensional continuous wavelet transform (cf. [9]). If we take *u*(*x*) as a local single-wave form *u*(*x*) = *ae* ^*ω*(*α*+*iβ*)·(*x*−*p*)^, then *W*(*u*; *p*, *σ*)(*ξ*) is expressed as

(6) W(u;p,σ)(ξ)=2πaσ2exp⁡[iω2σ2α·β+ω2σ2|α|22]×exp⁡[−i(p+ωσ2α)·ξ−σ2|ξ−ωβ|22].

Here we note that *ωβ* is a unique Gaussian peak of *W*(*u*; *p*, *σ*). The details of this derivation is given in the Appendix. The maximum arg⁡max⁡_*ξ*_ |*W*(*u*; *p*, *σ*)(*ξ*)| of the modulus |*W*(*u*; *p*, *σ*)(*ξ*)| for *ξ* ∈ ℝ^2^ is clearly achieved at *ξ*(*p*) = *ξ* = *ωβ*. Hence, we have

(7) $$\begin{array}{c} \beta =\underset{\xi }{\arg\;\max\;}\frac{\left|W\left(u;p,\sigma \right)\left(\xi \right)\right|}{\omega }. \end{array}$$

Here we note that *σ* ^2^ sitting in the denominator of the exponential of the Gaussian window will sit in the numerator of *W*(*u*; *p*, *σ*)(*ξ*). This is nothing but the Heisenberg uncertainty principle about the window sizes in the *real space x* and *Fourier space ξ*. We have an option to tune a parameter *σ* that influences the localization in the real space and Fourier space.

If *u*(*x*) is given as the multiple-wave form

(8) $$\begin{array}{c} u\left(x\right)=\sum_{n}c_{n}e^{\omega (\alpha_{n}+i\beta_{n})\cdot (x-p)},\;c_{n}\in \mathbb{C}, \end{array}$$

around *p*, arg⁡max⁡_*ξ*_ |*W*(*u*, *p*, *σ*)(*ξ*)| can expect to give the local wave vector *β*_*n*_ of the strongest single-wave form *c*_*n*_ *e* ^*ω*(*α*_*n*_+*iβ*_*n*_)·(*x*−*p*)^ in its modulus. This can be understood by accepting a very reasonable interpretation which says that the Gaussian peaks of the FBI-transformed *u* are well separated in most cases. We call this method to obtain the local wave vector *β*_*n*_ obtained above the local wave vector the LWV method.

**Figure 1:**
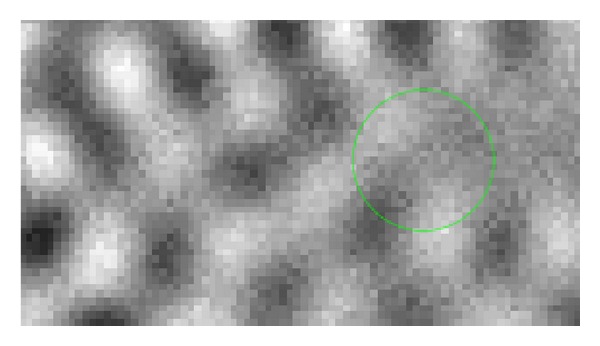
Example of function *Re*⁡*u*(*x*) of (8).

**Figure 2:**
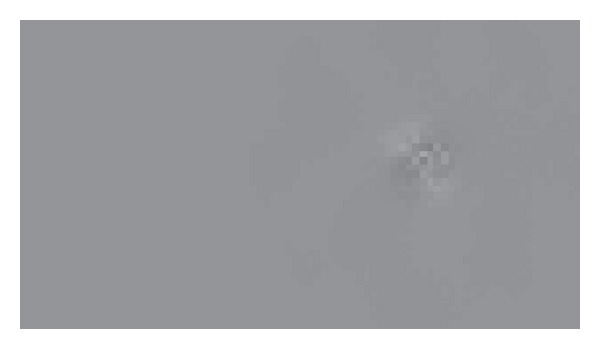
Localization of Figure 1 by Gaussian.

We will show in several figures how the LWV method is performed. Figures 1 and 2 show the localization by a Gaussian window. Since the key to the LWV method is the assumption that the local approximate expression of the wave *u* is given by (8), we need to localize *u* to find the local wave vector of *u*.

**Figure 3:**
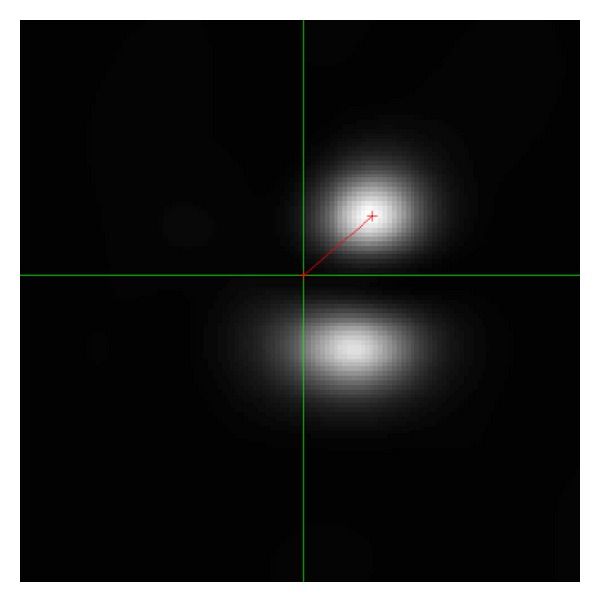
Example of |*W*(*u*; *p*, *σ*)(*ξ*)| in (7).

Figure 3 is what can be seen in the Fourier space *ξ*. More precisely this is the FBI-transformed image of Figure 2.

In this figure, we find two Gaussian peaks in Figure 3 which means that there are basically two different directions to which the waves are propagating in Figure 2. This reasonably fits to Figure 1. It seems that in the Fourier space, the position of the peak of Gaussian is not strongly interfered by those of other peaks of Gaussian. Hence, the separation of interfered waves in the Fourier space should be quite good.

**Figure 4:**
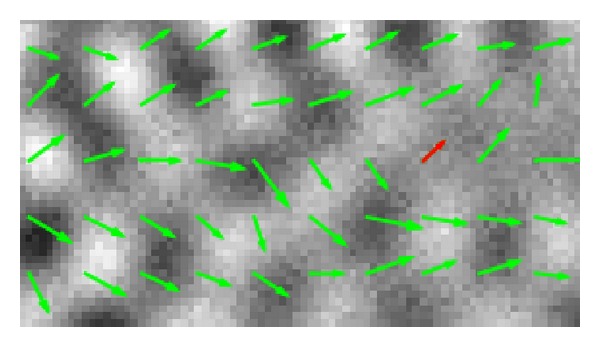
The vectors represent the wave vectors of the strongest waves. The red vector corresponds to that of Figure 3.

We repeated this process around enough sampled points and plotted the local wave vectors at the sampled points to obtain Figure 4 in which the sampled local wave vectors are superimposed over the figure of the real part of *u*.

Let us finish this section by giving several comments on the method. First of all, concerning the choice of the Gaussian window size *σ*, we usually take *σ* in the range from half wave length to one wave length for having reasonable recovery of *β* by our experiences. Taking arg⁡max⁡  may misfit *β* when there exists a strong noise with a specific frequency. But, for MRE data, it usually has only Gaussian-type white noise that does not have a specific frequency. Finally, we would like to emphasize here an advantage of the LWV method. That is, even in the case that several waves coming from different directions merge at a point *p* ∈ ℝ^2^, the effect of each wave is quite localized in the Fourier space, so that if there are several different waves merging at *p*, we can separate these major propagating directions by the LWV method.

## 3. LAV Method

We will show in this section how to recover the local attenuation vector of the strongest wave in the local multiple-wave form (8). To begin with we first assume that *u*(*x*) is given as a local single-wave form around a point *p* ∈ ℝ^2^. Then the vector *α* at *p* in the local single-wave form with the wave vector *β* can be recovered by

(9) $$\begin{array}{c} \alpha =-\frac{1}{\omega \sigma^{2}}\left(\nabla_{\xi }\theta \left(p;\xi \right)-p\right), \end{array}$$

where *θ*(*p*; *ξ*) is defined by

(10) θ(p;ξ):=arctan⁡(Im⁡⁡W(u;p,σ)(ξ)Re⁡W(u;p,σ)(ξ)).

In fact, substituting (6) into the right hand side of (9), introducing *θ*_0_ as an initial phase that does not depend of *ξ*, the right hand side of (10) becomes

(11) (RHS)=arctan⁡(sin⁡(−ωσ2α·ξ+θ0)cos⁡⁡(−ωσ2α·ξ+θ0))=arctan⁡(tan⁡(−ωσ2α·ξ+θ0))=−ωσ2α·ξ+θ0.

Then, we will obtain (9) by taking the gradient of *θ*(*p*; *ξ*) with respect to *ξ* at *ξ* = *ωβ* (Figure 6). In order to compute the gradient numerically we used the following least square method. Let *ξ* = (*ξ*_1_, *ξ*_2_),  *β* = (*β*_1_, *β*_2_) and denote *m* = *ξ*_1_ − *β*_1_,  *n* = *ξ*_2_ − *β*_2_. Then, the least square minimization to compute the gradient (∇_*ξ*_ *θ*)(*p*; *β*) is

(12) $$\begin{array}{c} \underset{\alpha_{1},\alpha_{2}}{\arg\;\min}\sum_{m,n}w\left(m,n\right){\left(\theta \left(p;\xi \right)-\theta \left(p;\beta \right)-\alpha_{1}m-\alpha_{2}n\right)}^{2}, \end{array}$$

where *w*(*m*, *n*) = *e* ^−(*m*^2^ + *n*^2^)/2*s*^2^^ with some constant *s* > 0.

**Figure 5:**
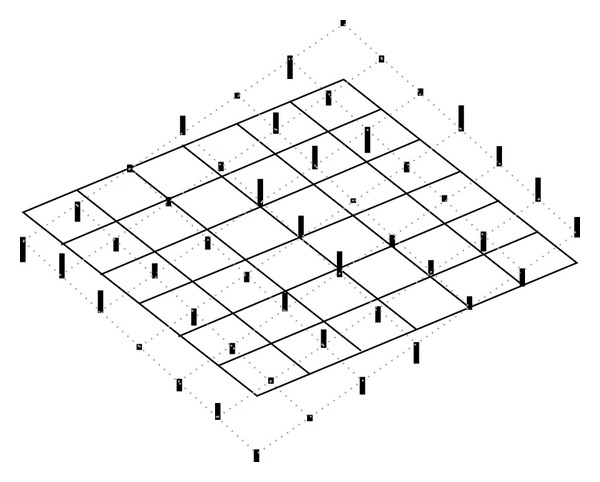
Least square method for 2-dimensional plane.

**Figure 6:**
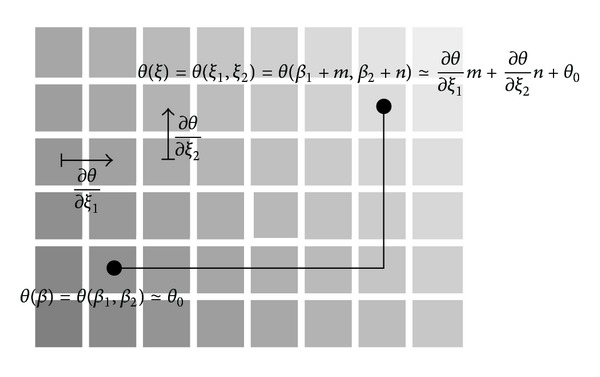
*θ* with respect to *ξ*.

Figure 5 illustrates the 3-dimensional view of this minimization.

Even for *u* having the local multiple-wave form, we apply the same formula (9) to compute the attenuation vector *α* associated with the local wave vector *β* by expecting that we have already picked up the strongest local single-wave form with the local wave vector *β* in the local multiple wave form and the contribution coming from the other local single-wave forms is small. This is the precise description of the LAV method.

In the rest of this section, we give a reminder for programming the LAV method. That is to handle the discontinuities of (10) at *θ* = ±*π*/2. Instead of using the formula

(13) θ(p;ξ)−θ(p;ξ0)=arctan⁡(Im⁡⁡W(u;p,σ)(ξ)Re⁡W(u;p,σ)(ξ))−arctan⁡(Im⁡⁡W(u;p,σ)(ξ0)Re⁡W(u;p,σ)(ξ0)),

we used as its reasonable approximation the following formula:

(14) θ(p;ξ)−θ(p;ξ0)=Im⁡⁡(W(u;p,σ)(ξ)W(u;p,σ)(ξ0)).

## 4. Numerical Testing of LWV and LAV Methods

In this section which consists of three subsections we will show some results on the numerical testing of our LWV and LAV methods. As we have mentioned before in Section 1, the methods are model-independent methods, but we will also show the numerical recoveries of *G*′,  *G*′′ in order to see the quantitative performance of our methods. The first subsection is for the numerical testing of our methods for simulated data and the succeeding two subsections are that for the real data obtained for phantoms by Mayo Clinic and MRE study group in our university, respectively. We call these real data the *phantom data* for simplicity. We did not test our methods for any clinical data, but the phantoms have some values close to the tissues of human levers.

### 4.1. Simulated Data

**Figure 7:**
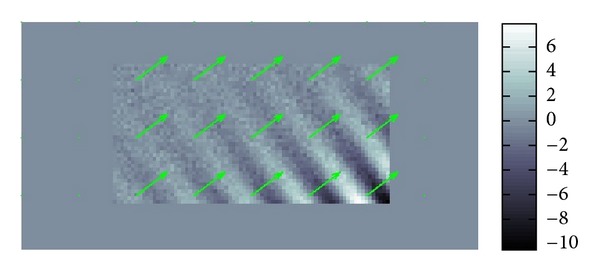
Recovered *β*.

**Figure 8:**
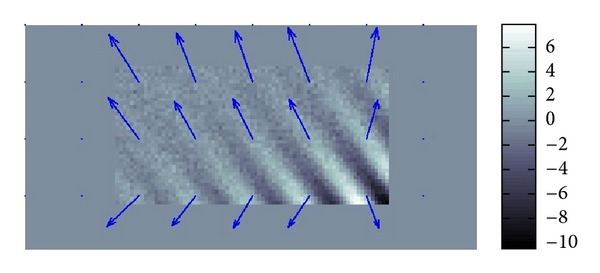
Recovered *α*.

For simulated data in an unbounded domain without any boundary and noise, the results of the numerical testing of our methods are perfectly fine. Hence, we will directly go to the numerical testing for simulated data in a bounded domain with boundary and a noise. We added a considerably large Gaussian-type noise to a simulated datum in order to see whether our methods work for the data with poor S/N ratio less than 0.1 which could be the case for real data. For the simulated datum, we made the length of *α* ten times longer than that of *β* which is the case for the phantoms data. Hence, the attenuation of wave is small. In other word, the amplitude of wave gradually decreases as the wave propagates. The superimposed arrows in Figures 7 and 8 show the recoveries of *β* and *α*. Hence, the variance of *α* in Figures 7 and 8 is smaller than it looks in Figure 8.

If there were no noise, then the recovered *α* and *β* should have been just constant vectors with the right upper direction and left upper direction for *β* and *α*, respectively. The recovery of *β* is quite good almost everywhere while that of *α* is less tolerant to noise and position.

**Figure 9:**
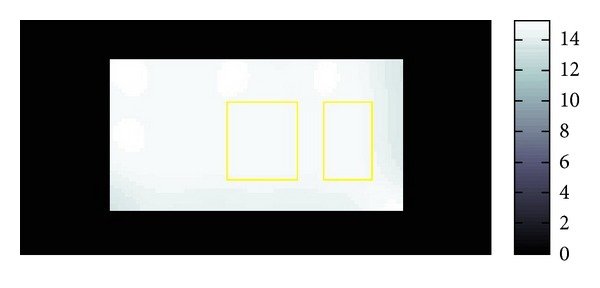
Distribution of *G*′ for simulated noisy data.

Next we computed *G*′ by using the formula (4).  Figure 9 shows the distribution of the value *G*′, and Table 1 shows the average and standard deviation of the distributed values of *G*′. We note that the true value of *G*′ was 14.4 kP. Hence, we can conclude from these that the recovery of *β* is quite good. We also observed by doing more numerical testing for simulated data that the estimate of *G*′ is always stable even under poor S/N ratio like this simulated data. Further we give two remarks. Firstly, for example, around the part of upper left corner of Figure 7, the signal is much less than background noise and hence we are nearly unable to see the pattern of waves there. Secondly, if *α* is much smaller than *β*, the simple approximate formula (cf. [10])

(15) $$\begin{array}{c} G^{\prime }=\frac{\rho }{{\left|\beta \right|}^{2}} \end{array}$$

of *G*′ works well.

**Figure 10:**
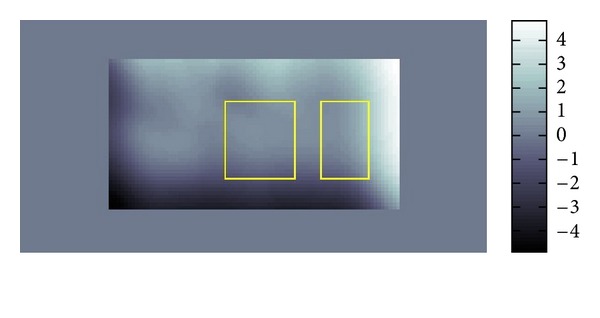
Distribution of *G*′′ for simulated noisy data.

We also computed *G*′′ by using the formula (4).  Figure 10 and Table 2 show the distribution of the value *G*′′ and the average value, standard deviation of the distributed values of *G*′′. These results show that the recovery of *G*′′ is not good in center, because expected value of *G*′′ is 0.69 kP. This insufficient recovery of *α* can be explained as follows. As we have seen before that the recovered *β* is almost a constant vector, but the recovered *α* fluctuates near the lower boundary almost completely changing its direction. Then, recalling the formula (4), the recovered *G*′′ is influenced by this fluctuation of *α* which can have negative sign. As far as we know, any MRE data analysis has a difficulty recovering *α* in an efficient way and we do have the same difficulty.

### 4.2. Phantom Data from Micro-MRE System

Now, we will show the testing of our method to a phantom datum obtained from MRE study group in Hokkaido University. The MRE system in Hokkaido University consists of micro MRI with a 0.3 tesla permanent magnetic, function generator and vibrating system. We call this MRE system the *micro-MRE system.*

**Figure 11:**
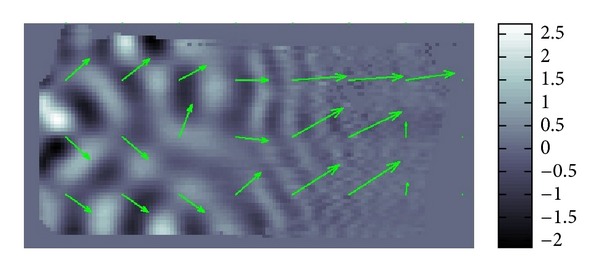
Fitting of *β*.

**Figure 12:**
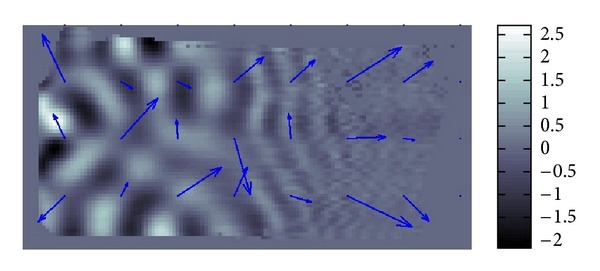
Fitting of *α*.

The resolution of the micro MRI is 1.2 mm square per pixel. The data obtained by this micro-MRE system for a phantom is given as the backgrounds of Figures 11 and 12 which are the same data for *Re*⁡*u*. The phantom is a two-layered PAAm gel and it has the cross section given as the rectangular region given in Figures 11 and 12 about 6 cm times 12 cm which is the plane containing the vibrating source. In this cross section, the location of the vibrating source is at the middle of the left edge and interface of the two layers appears in the middle. The left part of the cross section is stiffer than the right part. Also, the wave is generated from this source by the vibration system with the 250 Hz angular frequency and it travels to the right direction. The wave field looks much complicated than what we have seen before for the simulated simple sinusoidal wave and we can observe reflection and refraction of waves at the boundaries and interface, respectively.

We applied our method to recover *β* and *α*. The recovered *β* and *α* are shown in Figures 11 and 12. The result for *β* given in Figure 11 matches quite well the profile of the wave field. From Figure 12, we can see that the direction of *α* is not the same as direction of *β*. By plotting the modulus of *u*(*x*), that is, |*u*(*x*)|, we can observe that major waves, reflected waves, and transmitted waves are mixed together to yield standing waves which have small amplitude at some place and big amplitude antinode at other places creating some nodes. We can observe that *α* inclines to the nearest node.

**Figure 13:**
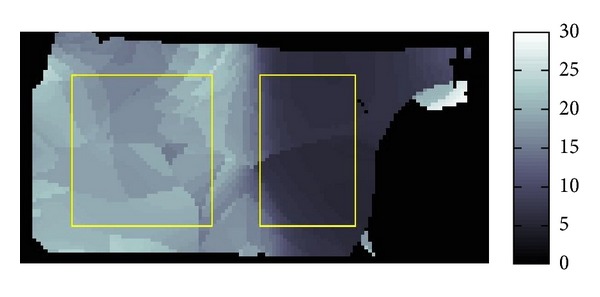
Distribution of *G*′ for two-layer phantom.

By the formula (4), we can transform the recovered *β*,  *α* into *G*′, *G*′′. The recovered *G*′ is given in Figure 13 and Table 3.

The *G*′ values of the two-layered phantom were also measured by a conventional rheometer giving the values 31.1 kPa and 10.7 kPa for the stiffer and softer parts of this phantom. The frequency of twisting the phantom was 10 Hz for this measurement. Since it is known that *G*′ depends on the frequency (cf. [11]), we cannot directly compare our result with these *G*′ values. The gray scale values in Figure 13 clearly show the location of the interface. Hence, we can say that our method can show the contrast of the stiffness. This is quite important in clinical application of MRE.

**Figure 14:**
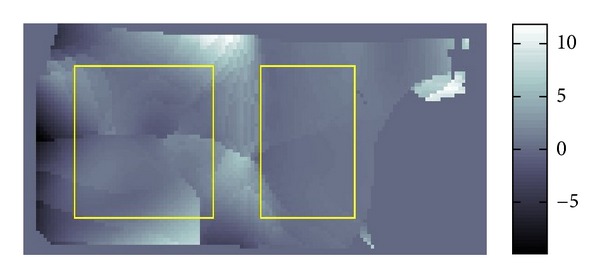
Distribution of *G*′′ for two-layer phantom.

Figure 14 and Table 4 show the recovered *G*′′.

Although we could recover *G*′′ to have a positive average value, comparing it with its standard deviation, the average value is smaller than its standard deviation. Looking more closer into the distribution of the recovered *G*′′  Figure 17, the average value in the center part is a small positive value, but there are some negative value in that part. Further the average value in the right part is uniformly positive which means that this value is reliable. As far as we know, our result is quite good compared with the other recovered values of *G*′′ by the direct method (cf. [12]) and modified integral method (cf. [3]). Nevertheless, we have to say that estimating the value of *G*′′ is not easy because it is a small value compared with the value of *G*′.

### 4.3. Data of Mayo Clinic

We used the data by courtesy of Mayo Clinic. From the attached information, the view is 20 cm square composed of 256 pixels each size. On the left side of the gel phantom, external vibration is continuously applied with 100 Hz sinusoidal displacement. The sample has four cylindrical inclusions and their diameters are 5, 10, 16, and 25 mm. The inclusions are stiffer than container. The original data have eight snapshots in 360 degrees phase shift. We altered the data into one complex-valued datum *u*(*x*) by using a weighted average for input of our method.

**Figure 15:**
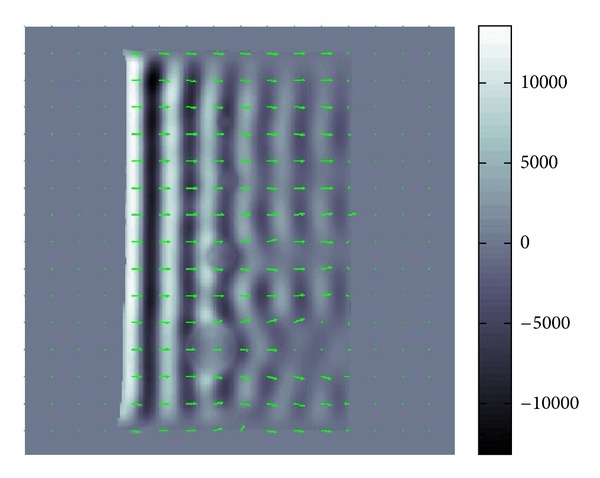
Fitting of *β*.

**Figure 16:**
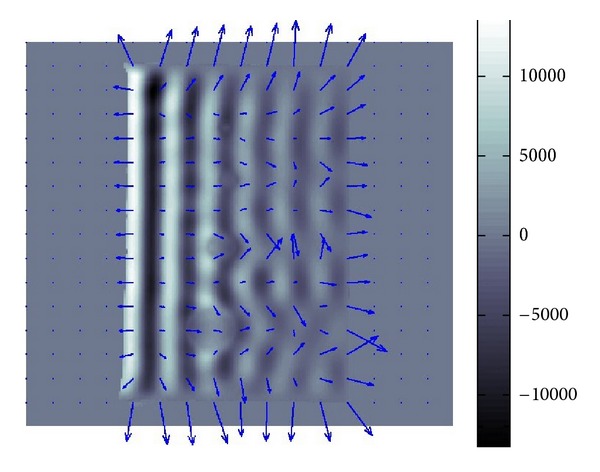
Fitting of *α*.

**Figure 17:**
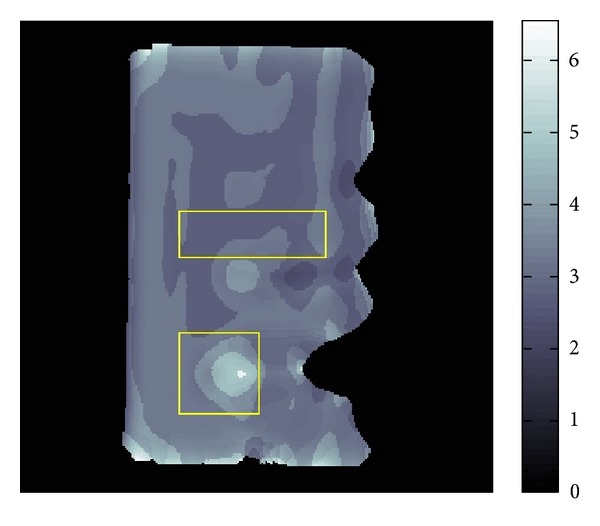
Distribution of *G*′ for Mayo Clinic data.

The original data is less noisy compared to our previous data. It is very near to the plain parallel wave except at the parts of inclusions. The vectors *β* in Figure 15 are nearly constant throughout the image. They are perturbed slightly near at the inclusions and their backsides.

The vectors *α* change their directions more sensibly than those of vectors *β*. The lengths of the vectors *α* are represented ten times longer than those of the vectors *β*. Therefore, an average, the values of *α* are smaller than those of *β*.

Table 5 shows that *G*′ in the bottom square is bigger than center. This gives the information that the inclusions are stiffer than the container. The value of standard deviation in bottom square is bigger than the another, because it nearly encloses the inclusion. On the result for the bottom square (Tables 3 and 6), we average *G*′ at a biggest inclusion and its neighborhood. If we take the area to be smaller, the estimated value of *G*′ goes higher. We compared our result with the result obtained by the modified integral method. The result by that method is believed to be stable and numerically reliable. It also supports our result of the average value because that output also has around 3.0 kP in the region without inclusion part.

The recovered result of *G*′ is not fully given. To be more specific, some part in the right hand side of the recovered result is intensionally cut off so that the result looks better. We have to explain why we did so. If *β* is zero or close to zero, we do not have any problem showing *β* as a vector. However in this case *G*′ will become so large, because *G*′ is proportional to |*β*|^−2^ by (15). This happens in the shadowed parts of the inclusions. In fact it is very difficult to see the nodal points of wave in these parts which could be coming from unsuccessful unwrapping of the MRE data, that is to specify the nodal value of wave in the MRE data. If there are not any nodal points in these parts, then the wave length there becomes infinitely long and hence the modulus of *β* will be very close to zero. This means that we could not trust the MRE data in these parts and this is why we cut off such parts.

For the recovered values of *G*′′, the ratio of *G*′′ to *G*′ fits the ratio which is commonly believed; that is, *G*′′ is about one-tenth order of *G*′.

## 5. Denoising and Sharpening

In this section, we will show that by a simple modification, the LWV method can be applied as a denoising for the MRE data. The principle behind this is as follows. For the local multiple-wave form *u* with *n* local single-wave forms, we have already observed that *W*(*u*; *p*, *σ*)(*ξ*) in most cases would have *n* well-separated Gaussian peaks. This can be used to filter the MRE data which denoises and sharpens the data.

### 5.1. LWV Denoising of MRE Data

Figure 19 is the whole view of Figure 4 which will be denoised.

The profile of waves is not so clear due to the noise. Our purpose here is to filter the data to reduce the noise and interferences of waves in the data shown by Figure 19.

Figure 20 shows the distribution of the modulus of *W*(*u*; *p*, *σ*)(*ξ*). From this we can know to which major directions the waves are propagating. Each Gaussian peak represents the major propagation direction for a certain group of waves. If these amplitudes of waves are large, then the peak becomes large also.

There are two ways to do the filtering. The one is to choose only the highest peak in the Fourier domain and remove the others. In detail, for each center point *p* of pixel, we replace *W*(*u*; *p*, *σ*)(*ξ*) by

(16) $$\begin{array}{c} W\left(u;p,\sigma \right)\left(\xi \right)\delta \left(\xi -\xi \left(p\right)\right), \end{array}$$

where *δ*(*ξ* − *ξ*(*p*)) is the delta function with a singularity at *ξ*(*p*) = arg⁡max⁡_*ξ*_ |*W*(*u*; *p*, *σ*)(*ξ*)|; that is, *ξ*(*p*) gives the position of the peak of |*W*(*u*; *p*, *σ*)(*ξ*)|, and then takes the inverse Fourier transform of (16) which is multiplied by the characteristic function of the aforementioned pixel. This process is done for each pixel and we obtain filtered waves by superposition. As a result we have Figure 21. We can tune denoising effect by replacing *δ*(*ξ* − *ξ*(*p*)) by a Gaussian window centered at *ξ*(*p*).

Figure 22 gives the modulus of the Fourier transformation of Figure 21 and we can see that there are two peaks. Also, as can be seen in Figure 21, there will be a discontinuity where two waves correspond to these peaks. Comparing Figures 20 and 22, we know that Figure 22 has much more clear and sharp images of waves.

Next, we will show another way of filtering. This is to filter the wave *u* around the globally strongest wave in the Fourier domain. That is let *ξ*_*∞*_ be the peak of the modulus of the Fourier transform of *u*. Then this filtering is to filter *u* in the previous way just around *ξ*_*∞*_ for each pixel. Then, we have Figures 23 and 24 for the filtered wave.

There is only a single wave which is close to a simple sinusoidal wave (Figure 23) and single peak in the Fourier domain (Figure 24).

### 5.2. Testing with Mayo Clinic Data

**Figure 18:**
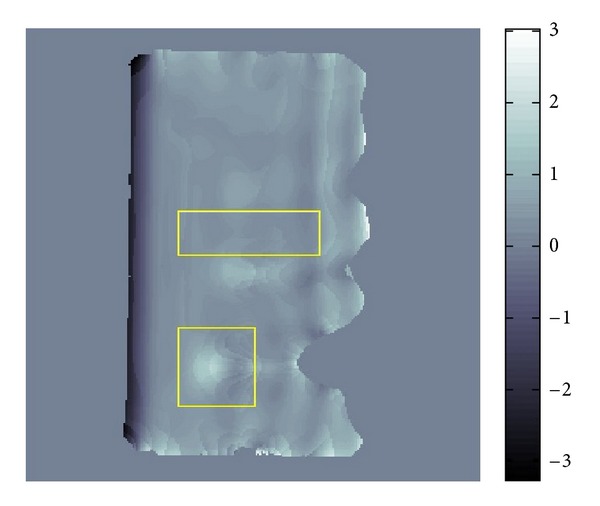
Distribution of *G*′′ for Mayo Clinic data.

**Figure 19:**
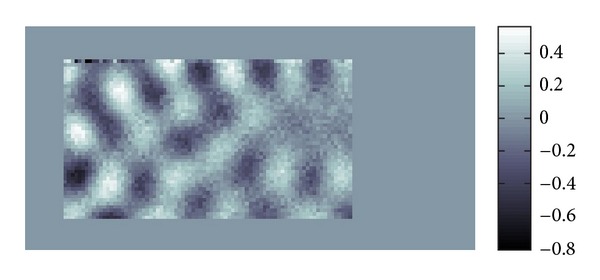
Real part in spatial domain.

**Figure 20:**
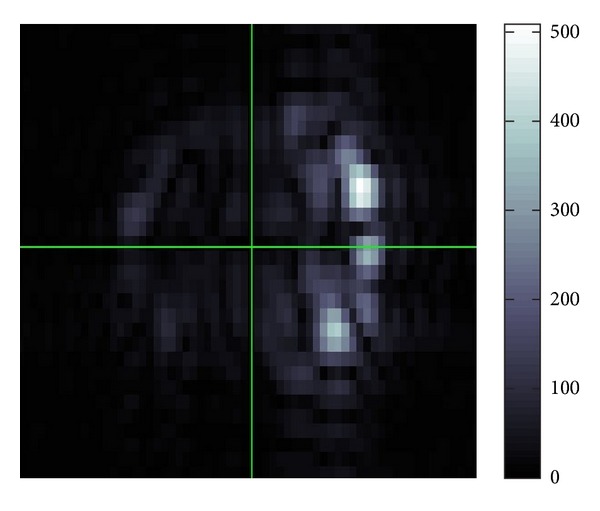
Modulus in Fourier domain.

**Figure 21:**
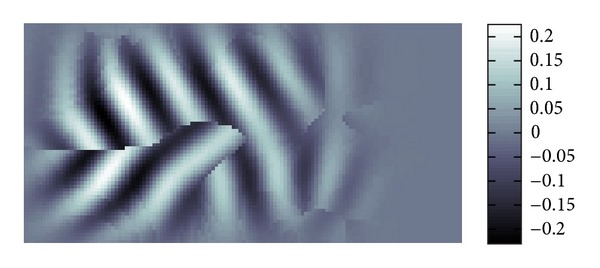
Real part in spatial domain.

**Figure 22:**
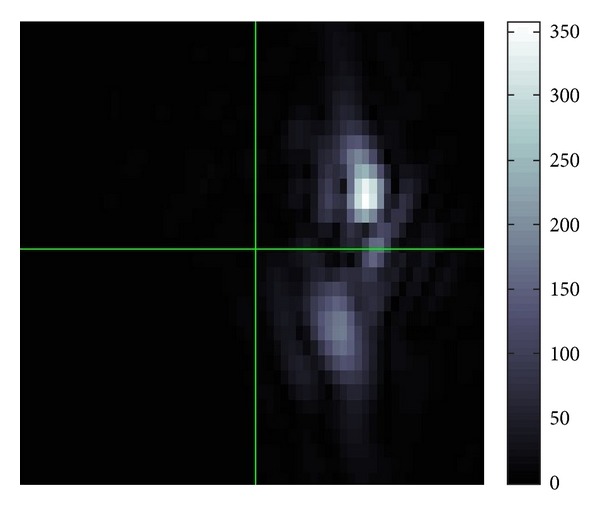
Modulus in Fourier domain.

**Figure 23:**
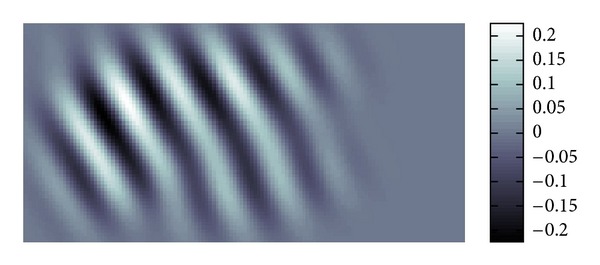
Real part in spatial domain.

**Figure 24:**
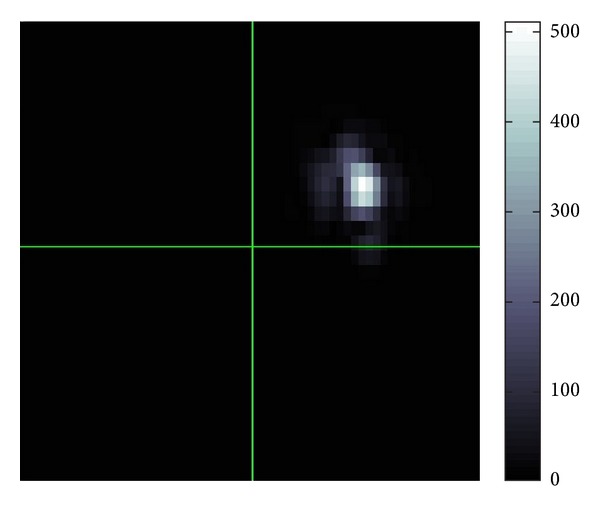
Modulus in Fourier domain.

**Figure 25:**
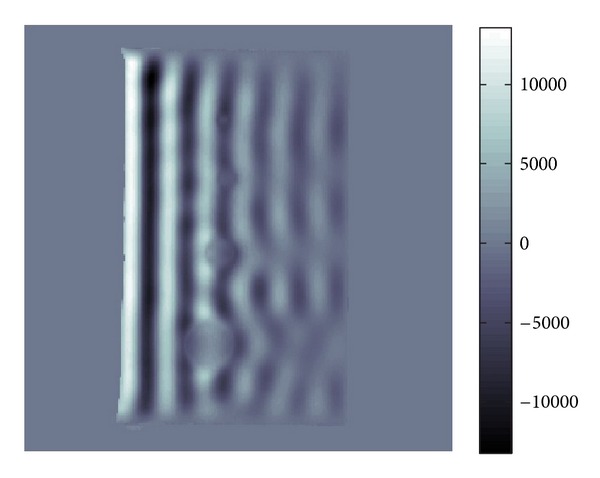
Data to be processed.

The LWV denoising always makes any data smooth taking off segmentations as well as noise in the data. Hence, if the input data is nearly free from noise, then the denoising process is unnecessary. For example, we applied the LWV denoising to the Mayo Clinic data (Figures 18 and 25).

**Figure 26:**
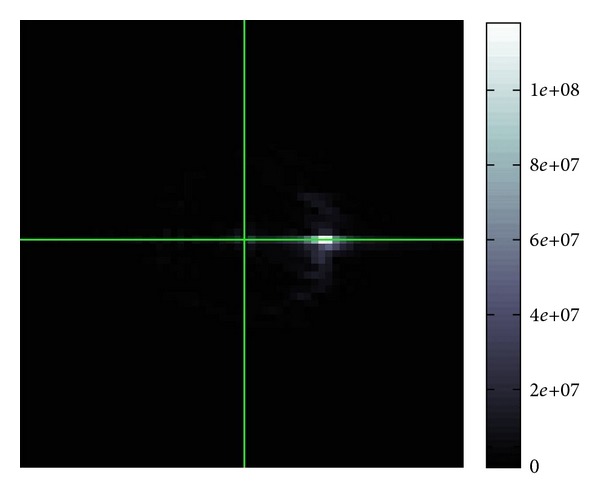
Modulus in Fourier domain.

**Figure 27:**
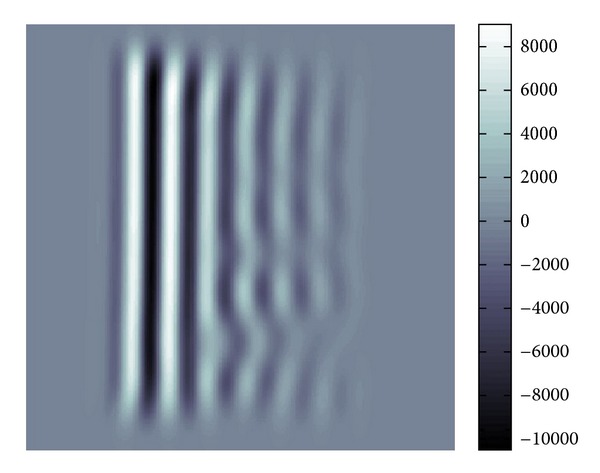
Filtering by the locally strongest wave.

**Figure 28:**
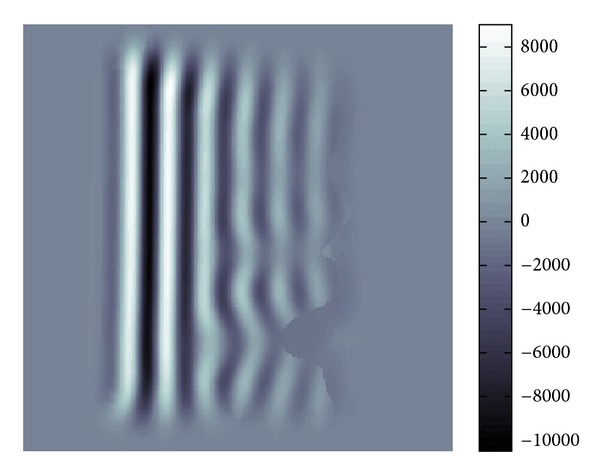
Filtering by the globally strongest wave.

Then, we obtained Figures 26, 27, and 28.

We can see that the denoising made the boundary of inclusions smoother and masked the inclusions.

## 6. Conclusions

We developed a model-independent data analysis for MRE data based on the FBI transformation to recover the local wave vector and local attenuation vector of the strongest local single wave assuming that waves in MRE data are locally given as a local multiwave form. This can be also applied to other wave images. We also linked the recovered local wave vector and local attenuation vector to the storage modulus and loss modulus by using a nearly incompressible isotropic viscoelastic equation which describes the displacement vector of time harmonic waves propagating in an MRE phantom. The recoveries of *β* and *G*′ were quite good and stable. Further, we showed that a modified version of LWV method which enables to recover the local wave vector can be used to denoise the MRE data.

Our MRE data analysis was conducted using a numerical computational software on Linux-based ordinary desktop computer. The fast Fourier transformation is not so time consuming for maximum 256 × 256 pixels MRE data. The overall calculation finished in order of minutes.

## Figures and Tables

**Table 1:** Estimation of *G*′.

Estimation of *G*′	Center	Right part
Average	14.89 kP	14.82 kP
Standard deviation	0.07 kP	0.11 kP

**Table 2:** Estimation of *G*′′.

Estimation of *G*′′	Center	Right part
Average	0.17 kP	0.70 kP
Standard deviation	0.59 kP	0.79 kP

**Table 3:** Estimation of *G*′.

Estimation of *G*′	Center	Right part
Average	18.56 kP	7.00 kP
Standard deviation	1.47 kP	1.83 kP

**Table 4:** Estimation of *G*′′.

Estimation of *G*′′	Center	Right part
Average	0.68 kP	0.60 kP
Standard deviation	1.05 kP	0.51 kP

**Table 5:** Estimation of *G*′.

Estimation of *G*′	Bottom square	Center
Average	3.71 kP	2.92 kP
Standard deviation	0.62 kP	0.24 kP

**Table 6:** Estimation of *G*′′.

Estimation of *G*′′	Bottom square	Center
Average	0.58 kP	0.34 kP
Standard deviation	0.36 kP	0.08 kP

## Appendix

### Fourier Transformation(FBI Transformation) of Waves

Let *u*(*x*) be of a single-wave form around a point *p* given by

(A.1) $$\begin{array}{c} u\left(x\right)=e^{\omega (\alpha +i\beta )\cdot (x-p)}, \end{array}$$

with *α*, *β* ∈ ℝ^2^. Then we compute its FBI transform as follows. By taking *x* − *p* as a new variable for the integration, we have

(A.2) W(u;p,σ)(ξ)=∫ℝ2eω(α+iβ)·(x−p)e−|x−p|2/2σ2e−ix·ξ dx=e−ip·ξ∫ℝ2eω(α+iβ)·xe−|x|2/2σ2e−ix·ξ dx.

Further, by *ωα* · *x* − (|*x*|^2^/2*σ* ^2^) = (*ω* ^2^ *σ* ^2^ | *α*|^2^/2)−(|*x* − *ωσ* ^2^ *α*|^2^/2*σ* ^2^) and taking *x* − *ωσ* ^2^ *α* as a new variable in the integration, we have

(A.3) ∫ℝ2eω(α+iβ)·xe−|x|2/2σ2e−ix·ξ dx =eω2σ2|α|2/2eiω2σ2α·βe−iωσ2α·ξ∫ℝ2eiωβ·xe−|x|2/2σ2e−ix·ξ dx.

Note that the integration in (A.3) is the Fourier transform of the Gaussian with respect to *ξ* − *β*. Hence,

(A.4) $$\begin{array}{c} \int_{{\mathbb{R}}^{2}}e^{i\omega \beta \cdot x}e^{-{\left|x\right|}^{2}/2\sigma^{2}}e^{-ix\cdot \xi }\;dx=2\pi \sigma^{2}e^{-\sigma^{2}{\left|\xi -\omega \beta \right|}^{2}/2}. \end{array}$$

After all, we have obtained

(A.5) W(u;p,σ)(ξ)=2πσ2exp⁡[iω2σ2α·β+ω2σ2|α|22]×exp⁡[−i(p+ωσ2α)·ξ−σ2|ξ−ωβ|22].
